# Studying the oxidation of water to molecular oxygen in photosynthetic and artificial systems by time-resolved membrane-inlet mass spectrometry

**DOI:** 10.3389/fpls.2013.00473

**Published:** 2013-11-26

**Authors:** Dmitriy Shevela, Johannes Messinger

**Affiliations:** Department of Chemistry, Chemistry Biology Centre, Umeå UniversityUmeå, Sweden

**Keywords:** isotope-ratio membrane-inlet mass spectrometry, isotope labeling, O_2_ evolution, photosynthetic and artificial water-splitting, photosystem II, water-oxidizing complex

## Abstract

Monitoring isotopic compositions of gaseous products (e.g., H_2_, O_2_, and CO_2_) by time-resolved isotope-ratio membrane-inlet mass spectrometry (TR-IR-MIMS) is widely used for kinetic and functional analyses in photosynthesis research. In particular, in combination with isotopic labeling, TR-MIMS became an essential and powerful research tool for the study of the mechanism of photosynthetic water-oxidation to molecular oxygen catalyzed by the water-oxidizing complex of photosystem II. Moreover, recently, the TR-MIMS and ^18^O-labeling approach was successfully applied for testing newly developed catalysts for artificial water-splitting and provided important insight about the mechanism and pathways of O_2_ formation. In this mini-review we summarize these results and provide a brief introduction into key aspects of the TR-MIMS technique and its perspectives for future studies of the enigmatic water-splitting chemistry.

## Introduction

**Figure 1:**
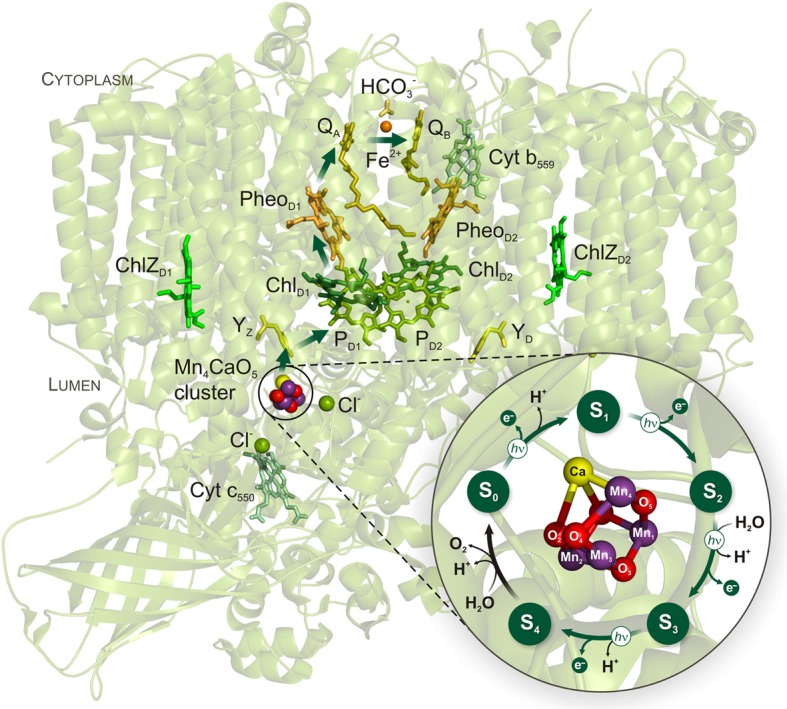
**Cyanobacterial PSII structure and Kok cycle of photosynthetic water oxidation by the Mn_4_CaO_5_ cluster**. The arrows within PSII indicate the direction of electron transfer which comprises the following redox-active cofactors: inorganic Mn_4_CaO_5_ cluster, redox-active tyrosine Z (Y_Z_), the primary electron donor P680 that includes a pair of Chls *a* (P_D1_ and P_D2_) and two accessory Chls (Chl_D1_ and Chl_D2_), the primary pheophytin (Pheo_D1_) acceptor, the primary (Q_A_) and the secondary (Q_B_) quinone acceptors. The phytyl tails of the Chl's and Pheo's, and the isoprenyl chains of the quinones have been cut for clarity. The light-induced S state transitions of the Mn_4_CaO_5_ cluster are indicated by arrows with “*h*ν” labels. The PSII structure and the zoomed structural model of the Mn_4_CaO_5_ cluster in the center of the Kok cycle are based on the recent PSII crystal structure at a resolution of 1.9 Å (PDB entry 3ARC; Umena et al., 2011).

In nature, the splitting of water to molecular oxygen (O_2_) is catalyzed by the membrane-bound pigment-protein photosystem II (PSII) of plants, algae, and cyanobacteria (Vinyard et al., 2013). The catalytic site of the water-splitting reaction is an inorganic tetra-manganese mono-calcium penta-oxygen (Mn_4_CaO_5_) cluster (Figure 1) that forms, together with its protein ligands, the water-oxidizing complex (WOC) of PSII (Yano et al., 2006; Umena et al., 2011). Water-splitting by the Mn_4_CaO_5_ cluster is energetically driven by the strongest biological oxidant, P680^+^ (with a midpoint potential of ~1.25 V), generated by the light-induced charge separation within the Chl-containing reaction center (RC) of PSII (Diner and Rappaport, 2002; Ishikita et al., 2005). A redox-active tyrosine residue (Y_Z_) is the essential electron transfer intermediate between the photoactive RC and the Mn_4_CaO_5_ cluster of PSII. Following light absorption, the Mn_4_CaO_5_ cluster is oxidized step-wise (one electron at a time) and thereby cycles through five redox states, known as S_*i*_ states (where *i* reflects the number of oxidizing equivalents stored by the cluster) (Figure 1). The four-electron four-proton oxidation chemistry of two water molecules is completed when the four oxidizing equivalents are accumulated within the WOC, and the highly reactive S_4_ state relaxes into the most reduced S_0_ state with the concomitant O–O bond formation and release of O_2_ (Messinger et al., 2012; Cox and Messinger, 2013). This reaction cycle of water oxidation is also known as the Kok cycle (Kok et al., 1970).

During the last few decades an enormous progress in elucidation of the WOC structure and in understanding the mechanism of the water-splitting became possible due to employment of numerous biophysical techniques (summarized in Aartsma and Matysik, 2008; Messinger et al., 2009a,b; also see refs therein). Among them, *time-resolved isotope-ratio membrane-inlet mass spectrometry* (TR-IR-MIMS) in combination with isotope labeling (Konermann et al., 2008; Beckmann et al., 2009) provided the most direct information on the S_*i*_ state dependent substrate water binding to the WOC (Messinger et al., 1995; Wydrzynski et al., 1996). These findings were recently reviewed in detail by Hillier and Wydrzynski (2008), Messinger et al. (2012), and Cox and Messinger (2013) and are, therefore, only briefly discussed here. However, TR-MIMS has also been successfully employed and yielded important data on other structural and mechanistic aspects of the water-splitting chemistry in both natural PSII and in variously designed artificial O_2_-evolving catalysts. In this mini-review, we summarize these recent investigations and also provide some comments on perspectives of the TR-MIMS technique for future studies of water-splitting and O_2_ evolution.

## Key concepts of TR-MIMS

The concept of TR-MIMS was first applied in 1963, when Georg Hoch and Bessel Kok began to use mass spectrometer with a *semipermeable membrane* as inlet system (Hoch and Kok, 1963). This allowed to separate the liquid sample from the high vacuum space of the mass spectrometer, while at the same time it was permeable to the gaseous analytes. This excellent solution allowed continuous *on-line* measurements of dissolved gaseous analytes (either dissolved in solution or directly from the gas phase) with a temporal resolution of a few seconds. Therefore, the TR-MIMS technique is ideally suited for investigations of photosynthetic and artificial water-oxidation/O_2_ evolution (for instance, see Konermann et al., 2008; Beckmann et al., 2009). For an outline of other TR-MIMS applications in biological and in industrial systems, see reviews by Lauritsen and Kotiaho (1996) and Johnson et al. (2000). Recent technological advances in MIMS instrumentation are summarized in Davey et al. (2011).

**Figure 2:**
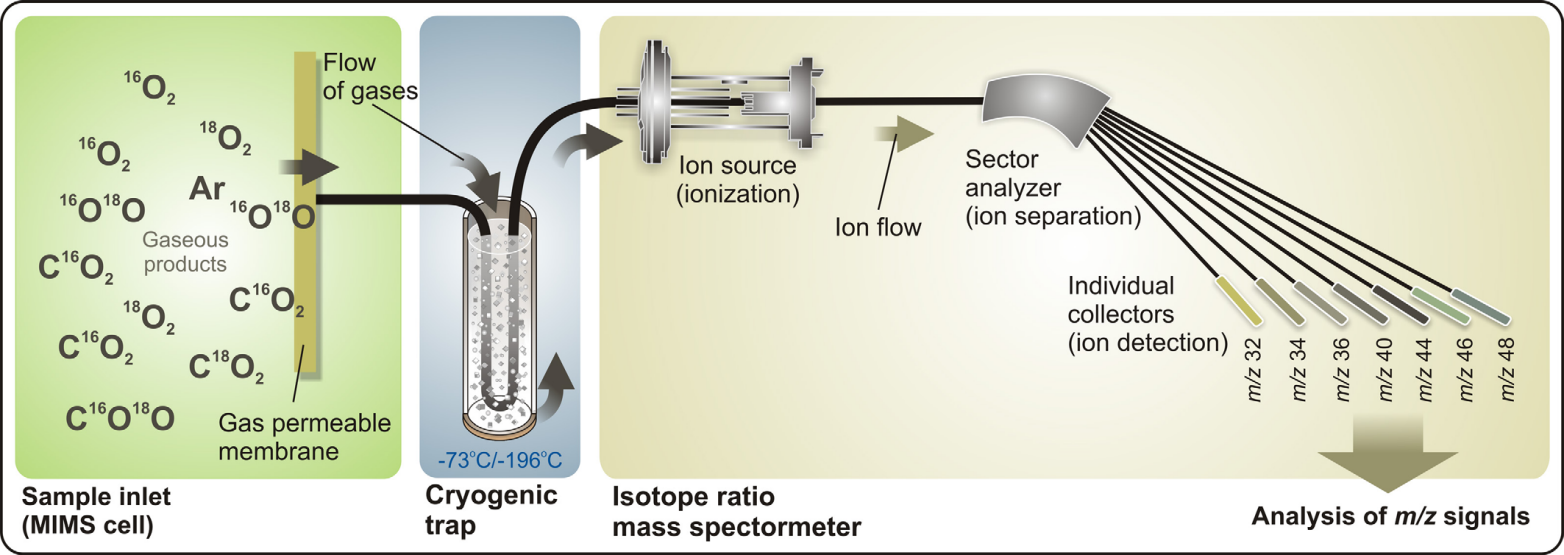
**Representation of a TR-IR-MIMS set-up**. Gaseous products, produced by sample suspension (for instance, by PSII samples) in the cell, penetrate through a gas-permeable membrane into a high-vacuum space, pass through a cryogenic trap (which removes water vapor from a flow of gaseous analytes), and enter the isotope ratio mass spectrometer. Here, gaseous analytes are first ionized in the ion source by electron impact, and are then separated according to their *m/z* ratios by a magnetic field in the sector analyzer that allows simultaneous online detection by individual collector cups (e.g., a 7-cup Faraday detector array). MS signals are monitored and analyzed using a personal computer. See text for further details.

A schematic view of a TR-MIMS set-up employing an isotope ratio mass spectrometer is shown in Figure 2. This type of mass spectrometers is normally equipped with an electron-impact ion source, magnetic sector field analyzer, and individual detectors (Faraday cups) that provide simultaneous detection of several masses (ions) with high sensitivity and signal stability. For its ability to monitor and to selectively analyze all *isotopologues* (molecules that differ only in their isotopic composition) of gaseous products with one instrument, the TR-MIMS approach in combination with isotope enrichments became indispensable tool for kinetic and functional analyses of photosynthetic enzymes (Konermann et al., 2008; Beckmann et al., 2009). The key part of the TR-MIMS instrument is a gas inlet system that is integrated within a MIMS cell. The design of MIMS cells may vary depending on the measuring purposes (Konermann et al., 2008; Beckmann et al., 2009), but all of them contain a gas-permeable membrane functioning as analyte inlet system into the vacuum of the mass spectrometer. The coupling of such a cell to various light sources (e.g., Xenon lamps or lasers) allows carrying out the measurements of light-induced O_2_ evolution in photosynthetic samples or light-driven O_2_-evolving artificial catalysts. Before entering the ion source of the mass spectrometer the analytes pass through a cryogenic trap (Figure 2), which freezes out water vapor that inadvertently pervaporate through the membrane in trace amounts.

Enrichment of aqueous sample suspension with oxygen's heavy isotope (^18^O) for isotope ratio measurements of O_2_ (and/or CO_2_) isotopologues is a powerful and commonly used tool in studies of water-splitting chemistry and/or related reactions. Therefore, most of the experiments are carried out in H^18^_2_O-labeled sample suspensions/solutions.

## Is water the immediate substrate of photosynthetic O_2_ evolution?

It is widely accepted that water is the immediate substrate for photosynthetic O_2_ production. However, Metzner (1978) suggested that instead hydrogen carbonate (bicarbonate; HCO^−^_3_) is the immediate substrate for O_2_ formation that is subsequently replenished by the reaction of CO_2_ with H_2_O. This hypothesis was discounted for long because the isotopic equilibration between ^18^O-water and HCO^−^_3_ is too slow to account for early isotope labeling studies (Ruben et al., 1941; Stemler and Radmer, 1975; Stevens et al., 1975; Radmer and Ollinger, 1980). Due to the discovery that a carbonic anhydrase (CA) activity is associated with PSII (Lu and Stemler, 2002; Villarejo et al., 2002; Moskvin et al., 2004) the “bicarbonate-as-substrate hypothesis” needed to be re-investigated with refined expriments. Indeed, due to rapid exchange of HCO^−^_3_ and CO_2_ species by CA, the ^18^O-label could “escape” from HCO^−^_3_ to water (which has a several orders higher concentration than the added ^18^O-labeled HCO^−^_3_), and, thus, lead to the lack of O_2_ yield from HCO^−^_3_ (Stemler and Radmer, 1975; Radmer and Ollinger, 1980).

Two different TR-MIMS approaches were taken recently and both exclude that HCO^−^_3_ is a physiologically significant substrate (Clausen et al., 2005a; Hillier et al., 2006). Clausen et al. (2005a) reported that under H^18^_2_O-labeled and CO_2_/HCO^−^_3_-depleted conditions the typical oscillation pattern of ^18^O-enriched O_2_ evolution is obtained in response to single light flashes, but didn't find any evidence for CO_2_ release. The latter would be expected in case the Metzner's hypothesis would be correct. Hillier et al. (2006), in their TR-MIMS study, employed ^18^O/^13^C-labeled HCO^−^_3_ to probe the capability of PSII (from higher plants and cyanobacteria) to oxidize HCO^−^_3_. The authors were able to detect an extremely small (and, thus, non-physiological) flux of ^18^O from HCO^−^_3_ into O_2_ similar to that observed in an early TR-MIMS study of Radmer and Ollinger (1980). Moreover, no relationship between O_2_ evolution and PSII-associated CA activity was found by McConnell et al. (2007) in their TR-MIMS examination of PSII preparations from higher plants.

## Is hydrogen carbonate a ligand to the WOC?

Hydrogen carbonate had been proposed to bind as integral cofactor to the Mn_4_CaO_5_ cluster after accumulation of many experimental results indicating: (i) the requirement of HCO^−^_3_ ions for optimal stability and functionality of the WOC (Van Rensen and Klimov, 2005), and (ii) it's important role for the photoassembly reaction of the Mn_4_CaO_5_ cluster (Dasgupta et al., 2008). Moreover, in the PSII crystal structure from *Thermosynechococcus elongatus* at 3.5 Å resolution, HCO^−^_3_ was modeled as a non-protein ligand bridging Mn and Ca ions within the WOC (Ferreira et al., 2004).

Earlier, interesting TR-MIMS experiments were performed by Stemler (1989) and Govindjee et al. (1991), in which formate was tested to induce the release of HCO^−^_3_ (that can be detected by TR-MIMS as CO_2_) from PSII. Although Govindjee et al. (1991) provided clear evidence for the formate-induced release of CO_2_/HCO^−^_3_, the HCO^−^_3_ binding site was not specified in this study. Based on numerous previous data indicating that HCO^−^_3_ is a ligand to the non-heme iron (NHI) at the electron acceptor side of PSII, the released CO_2_ was suggested to derive from this binding site. However, later formate was shown to bind both at the acceptor and the donor (water-splitting) side of PSII (Feyziev et al., 2000), and therefore, the released CO_2_/HCO^−^_3_ could also originate from the water-splitting side.

**Figure 3:**
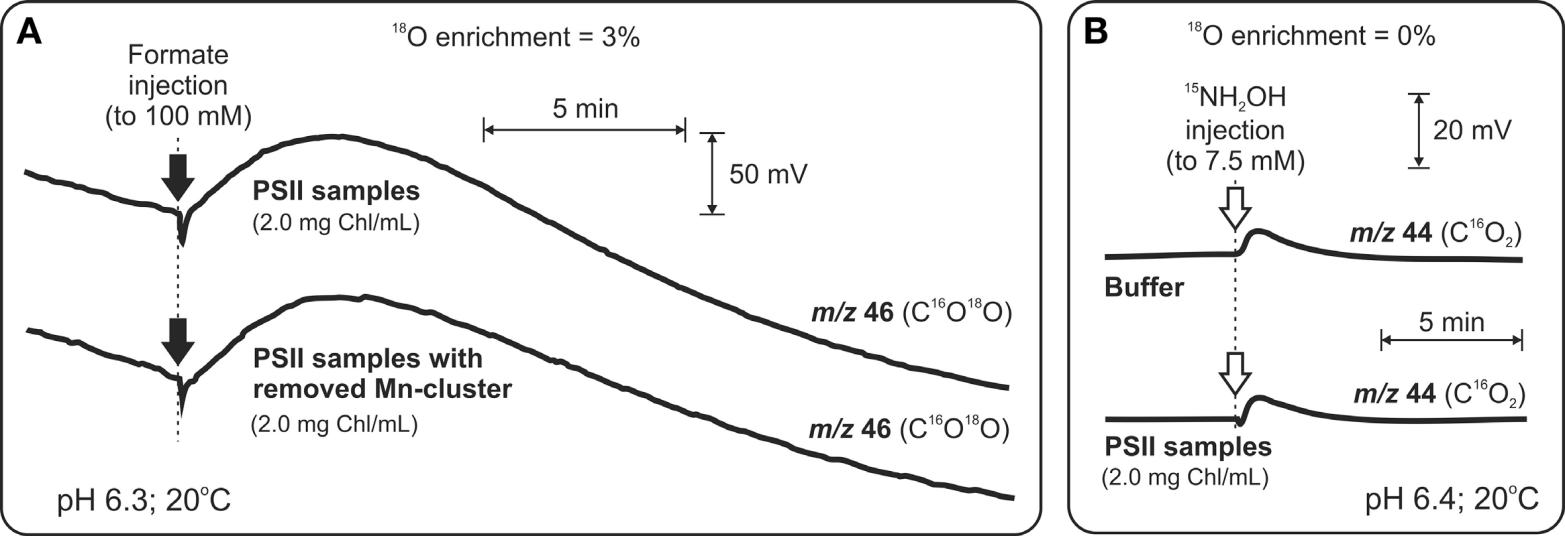
**TR-MIMS experiments demonstrating that HCO^−^_3_ is not a tightly bound ligand to the Mn_4_CaO_5_ cluster in spinach PSII membrane fragments. (A)** Amount of released CO_2_ upon formate addition (black arrows) to intact PSII membranes is the same as in the case of PSII membranes without the Mn_4_CaO_5_ cluster (due to 75-min pre-incubation with 80 mM N_2_H_4_). Due to enrichment of sample suspension with H^18^_2_O (3%) CO_2_ was detected as C^16^O^18^O at *m/z* 46. **(B)** Addition of the strong reductant NH_2_OH (white arrows) at concentrations known to cause rapid reduction of the Mn_4_CaO_5_ cluster and release of Mn ions as Mn^II^ into the solution didn't lead to a release of CO_2_/HCO^−^_3_ above background. In order to avoid the overlay of CO_2_ and N_2_O signals (the latter is known to be produced during interaction of NH_2_OH with the Mn_4_CaO_4_ cluster), the N_2_O signal was shifted from *m/z* 44 to *m/z* 46 by employing the ^15^N-labeled NH_2_OH for these experiments. To facilitate equilibration between CO_2_ and HCO^−^_3_ all measurements were performed in the presence of externally added CA (to a final concentration of 3 μg ml^−1^). Modified from Shevela et al. (2008b).

In order to specifically probe the possible binding of HCO^−^_3_ to the Mn_4_CaO_5_ cluster at the donor side of PSII, Shevela et al. (2008a,b) re-examined and extended the earlier TR-MIMS investigations. Thus, a comparison of the formate-induced C^16^O^18^O yields (Figure 3A), under H^18^_2_O-enriched conditions, in intact PSII and Mn-depleted PSII was performed. This was complemented by experiments in which the gaseous products produced by a quick reductive destruction of the of the Mn_4_CaO_5_ cluster by ^15^N-labeleld NH_2_OH (Figure 3B) were studied. Both approaches clearly demonstrated that the detected CO_2_/HCO^−^_3_ *does not* originate from the inorganic core of the water-splitting site of PSII (Shevela et al., 2008a,b). Independent evidence for absence of HCO^−^_3_ bound to the WOC was provided by FTIR and GS-MS experiments (Aoyama et al., 2008; Ulas et al., 2008). Moreover, in recent x-ray crystallography studies at resolutions of 1.9-3.0 Å no HCO^−^_3_ was found in the vicinity of the Mn_4_CaO_5_ cluster, while they all clearly show HCO^−^_3_ bound to the NHI on the acceptor side of PSII (Guskov et al., 2010; Umena et al., 2011) (also, see Figure 1). Thus, it can be excluded that HCO^−^_3_ is a tightly bound ligand of the Mn_4_CaO_5_ cluster.

However, none of the mentioned studies negates the option that a mobile, weakly bound, and rapidly exchanging HCO^−^_3_ affects the activity of the WOC. Thus, in case of the TR-MIMS measurements, weakly bound HCO^−^_3_ may have been removed during the required degassing of the MIMS cell prior to formate or NH_2_OH additions to PSII samples. Therefore, the possible loss of weakly bound HCO^−^_3_ and the amount of HCO^−^_3_ associated with PSII under air-saturated condition remain to be established in future TR-MIMS experiments.

## When and how does substrate water bind to the WOC?

**Figure 4:**
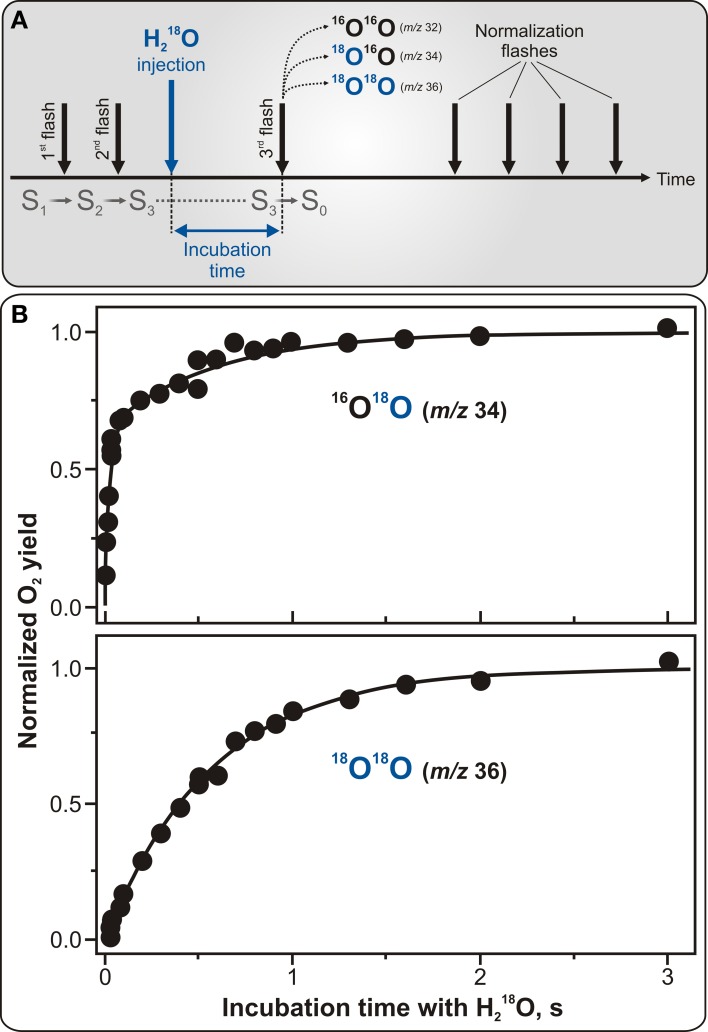
**Protocol for TR-MIMS measurements of H_2_^16^O/H^18^_2_O exchange in the S_3_ state of PSII (A) and experimentally obtained substrate water exchange rates in spinach thylakoids (B). (A)** The S_3_ state is populated by two pre-flashes given at 2 Hz (shown by the two first black vertical arrows). This is followed by the rapid injection of H^18^_2_O into the PSII sample (shown by blue vertical arrow) and subsequent fast mixing of the injected H^18^_2_O with the sample. Evolution of O_2_ isotopologues is then induced by a 3rd flash, given at varying delay times (from 0 to 10 s) after the H^18^_2_O injection (signified as incubation time). Finally, a series of four flashes is given at 2 Hz to induce O_2_ yield used for normalization. **(B)** TR-MIMS measurements of substrate H_2_^16^O/H^18^_2_O exchange kinetics were performed at *m/z* 34 (top plot) for singly-labeled isotopologue ^16^O^18^O, and at *m/z* 36 (bottom plot) for doubly-labeled ^18^O^18^O in the S_3_ state in spinach thylakoids at 10°C and pH 6.8. Symbols in both plots are experimental data, and the lines in the top and bottom plots are biexponential and monoexponential fits, respectively. The biexponential fit yields rate constants of ~40 s^−1^ for the fast phase and ~2 s^−1^ for the slow phase. The slow phase in the ^16^O^18^O data is matching the rate found in the monoexponential fit of the ^18^O^18^O data (Messinger et al., 1995; Hillier et al., 1998; Hillier and Wydrzynski, 2000, 2004). Adapted from Cox and Messinger (2013).

Indisputably, the most significant and unique contribution of the TR-MIMS instrumentation in understanding of water-oxidation mechanism in photosynthesis was its application for studying substrate binding in the different S_*i*_ states of the WOC. In these experiments the binding of water to the WOC is probed by the rapid injection of H^18^_2_O into the PSII samples which were preset into the desired S_*i*_ state by pre-illumination with 0, 1, 2, or 3 flashes. Then, after desired incubation times, O_2_ evolution is induced by a sequence of additional flashes. The exchange rates of the two substrate molecules are then calculated from the change of the ^16^O^18^O and ^18^O^18^O yields as a function of incubation time (see Figure 4A for protocol of the TR-MIMS measurements of substrate water exchange in the S_3_ state). The mixing time of H^18^_2_O with the PSII samples after injection and a very low level of dissolved O_2_ in H^18^_2_O are highly important for these experiments since they determine the time resolution of the TR-MIMS measurements. In the first H_2_^16^O/H^18^_2_O-exchange TR-MIMS experiments the water exchange kinetics could not be resolved (Radmer and Ollinger, 1986; Bader et al., 1993). The development of the MIMS cell by Messinger et al. (1995), which allowed for fast mixing of H^18^_2_O with the sample and also implemented O_2_ removal from the labeled water by the glucose—glucose oxidase—catalase method, greatly improved the time resolution down to the milliseconds scale and allowed measurements of substrate water exchange in all semistable S_*i*_ states (Messinger et al., 1995; Hillier et al., 1998; Hillier and Wydrzynski, 2000).

Figure 4B illustrates characteristic water exchange kinetics in the S_3_ state as measured in spinach thylakoids with the time resolution of 8 ms. In this figure, the yields of the singly-labeled (^16^O^18^O) and doubly-labeled (^18^O^18^O) isotopologues of molecular oxygen are plotted as a function of H^18^_2_O incubation time in the S_3_ state. While the former plot reflects the result when only one of the two possible ^18^O-water substrates is exchanged, the latter one is for the case when both ^18^O-waters are exchanged. The biphasic behavior of the ^16^O^18^O rise (detected at *m/z* 34) (see Figure 4B) is known to represent the exchange rates of two independent *slowly* and *fast* exchanging substrate water molecules bound at separate sites within the WOC. In contrast, the ^18^O^18^O product (monitored at *m/z* 36) exhibits a mono-exponential rise with a rate equal to that of the slow phase kinetic of the ^16^O^18^O data, thus-reflecting the exchange of the same “slowly” exchanging substrate water as observed at *m/z* 34. This finding clearly confirms that the two phases of the ^16^O^18^O data are an intrinsic feature of the WOC and do not originate from PSII heterogeneity (Messinger et al., 1995; Hillier et al., 1998).

Further TR-MIMS experiments also revealed that the “slowly” exchanging water is bound to the WOC in all semi-stable S_*i*_ states, while the “fast” exchanging water was detected only in the S_2_ and S_3_ states (Hillier et al., 1998; Hillier and Wydrzynski, 2000, 2004; Hendry and Wydrzynski, 2002). Thus, the TR-MIMS technique provides not only the most direct evidence for independent substrate water binding within the WOC, but also allows to monitor the change in their binding affinities throughout the reaction cycle. For a complete overview of the TR-MIMS findings in this field, we refer the readers to reviews by Hillier and Messinger (2005), Hillier and Wydrzynski (2008), Beckmann et al. (2009), Messinger et al. (2012), and Cox and Messinger (2013).

## The ^16^O/^18^O isotope effect and photosynthetic water-splitting

Up to now there is no final agreement on whether isotopic discrimination during O_2_ production by photosynthetic water-splitting in PSII contributes to the so-called *Dole effect*, which describes the finding that the percentage of the ^18^O isotope in atmosphere is higher (by 23‰) than in oceanic waters (Dole, 1936; Tcherkez and Farquhar, 2007). While many gas isotope ratio studies clearly showed that oxygen produced by O_2_-evolving organisms is isotopically identical to the water they are suspended in (Dole and Jenks, 1944; Stevens et al., 1975; Guy et al., 1993; Helman et al., 2005), recent ^18^O-enriched TR-MIMS experiments indicated that the ^18^O isotope is favored by the WOC for O_2_ production, thus-suggesting a significant ^16^O/^18^O isotope effect in the photosynthetic water-splitting (Burda et al., 2001, 2003). This finding was challenged by recent theoretical estimations that suggest a very small isotope effect (Tcherkez and Farquhar, 2007). Undoubtedly, a resolution of these conflicting results can be provided by revisiting TR-MIMS studies. These future studies should be designed to account for: (i) technical limitations/drawbacks of the previous TR-MIMS experiments (for instance, the absence of fast H^18^_2_O mixing upon its addition to sample suspension inside the MIMS cuvette Bader et al., 1987; Burda et al., 2003), (ii) possible contribution of isotopic fractionation due to transfer of O_2_ isotopologues through the membrane inlet toward the high vacuum of mass spectrometer recently reported by Hillier et al. (2006), and (iii) current knowledge of the S-state dependent substrate water binding and exchange rates as derived from TR-MIMS measurements (for reviews, see Hillier and Wydrzynski, 2008; Cox and Messinger, 2013). However, we note here, that without specific investigations of the ^18^O isotope effect in photosynthetic water-oxidation, our TR-MIMS studies do not reveal any oxygen isotope discrimination in photosynthetically produced O_2_ (for instance, see Figure 6C and text below for explanations), indicating that any such effect must be small at best.

## In search for intermediates of water splitting by TR-MIMS approach

**Figure 5:**
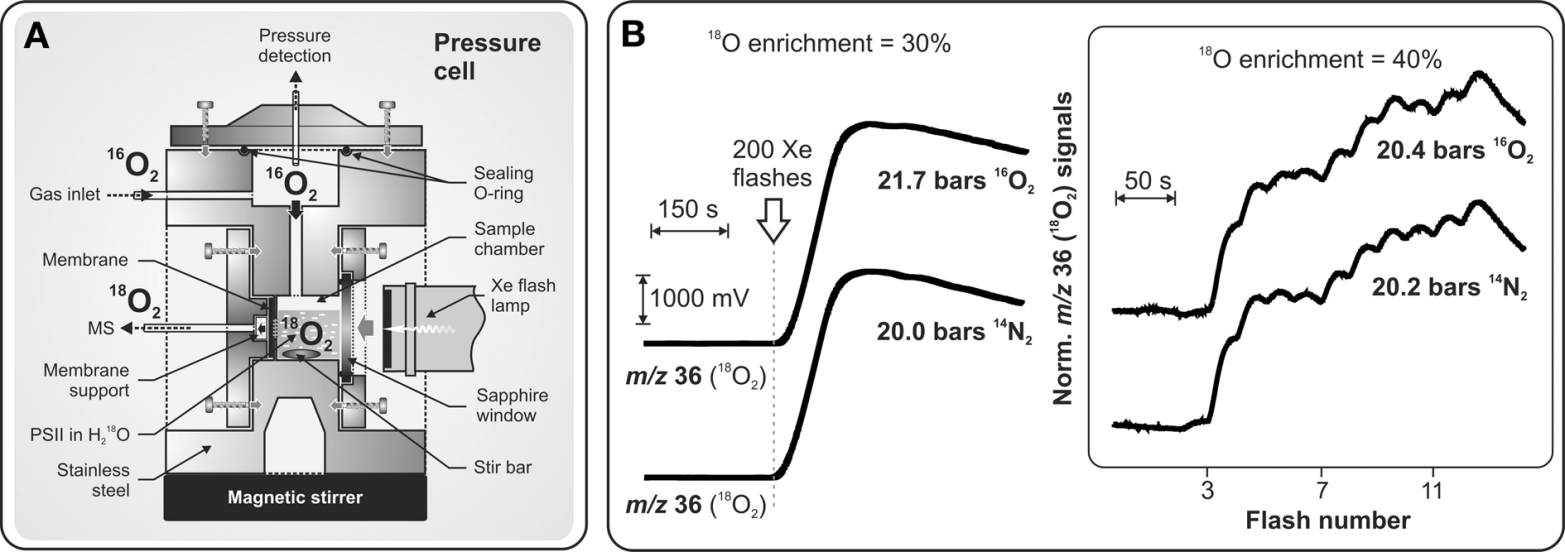
**Schematic representation of the pressure cell (A) specially designed for TR-MIMS measurements of light-induced ^18^O_2_ evolution of PSII under high ^16^O_2_/N_2_ pressures (up to 20 bars). (B) *MIMS* signals in panel (B)**; **Left**: ^18^O_2_ production of PSII core complexes from *Synechocystis* sp. PCC 6803 induced by a series of 200 saturating Xenon flashes (given at 2 Hz; indicated by arrow) at 21.7 bars O_2_, or 20 bars N_2_. Other conditions: 30% H^18^_2_O enrichment; [Chl] = 50 μM; 250 μM DCBQ, pH 6.7, 20°C. **Right:** Flash-induced ^18^O_2_ evolution patterns of PSII membrane fragments from spinach induced by a series of saturating laser flashes (separated by dark times of 25 s) at 20.4 bars O_2_, or 20.2 bars N_2_. Other conditions: as above, but with 40% H^18^_2_O. Adapted from Shevela et al. (2011a).

While most states of the Kok cycle (S_0_, S_1_, S_2_, S_3_) are semistable, the S_3_Y^•^_Z_ and S_4_ state are known to be a highly reactive intermediates that until very recently were not characterized. Clausen and Junge (2004) attempted to stabilize and identify putative intermediate(s) of the S_4_ state by applying a high partial O_2_ pressure in order to shift the equilibrium of the terminal S_4_ → S_0_ + O_2_ + *n*H^+^ reaction backwards. Based on their UV-absorption transients the authors observed half suppression of Mn oxidation under only 10-fold increase of ambient O_2_ pressure (2.3 bar). These results were considered to be the first indication for an intermediate in the S_3_ → S_4_ → S_0_ transition and as a possible route for stabilizing it (Clausen and Junge, 2004). Although a further delayed Chl fluorescence study corroborated these results (Clausen et al., 2005b), experiments by time-resolved X-ray absorption spectroscopy (TR-XAS) (Haumann et al., 2008) and by visible fluorescence study (Kolling et al., 2009) shed doubt on the existence of accessible S_4_ intermediate(s) that can be populated by inhibition of the terminal step of O_2_ release from the WOC by elevated O_2_ concentrations. These controversial studies prompted application of the TR-MIMS technique, which allowed investigation of the effect of elevated O_2_ pressure on photosynthetic O_2_ release by direct O_2_ detection (Shevela et al., 2011a). In these experiments direct monitoring of ^18^O_2_ evolution from ^18^O-labeled water against a high level of ^16^O_2_ in a suspension of PSII complexes became possible due to a specially designed high pressure MIMS cell (for details, see Figure 5A). This study demonstrated that neither an inhibition nor altered flash-induced pattern of O_2_ evolution take place under up to 50-fold increased concentration of dissolved O_2_ around PSII (Figure 5B). These findings show that the terminal water-splitting reaction/O_2_ release in PSII is highly exothermic, and are in line with the results obtained by TR-XAS (Haumann et al., 2008) and variable fluorescence (Kolling et al., 2009) studies.

## Applications in artificial photosynthesis

**Figure 6:**
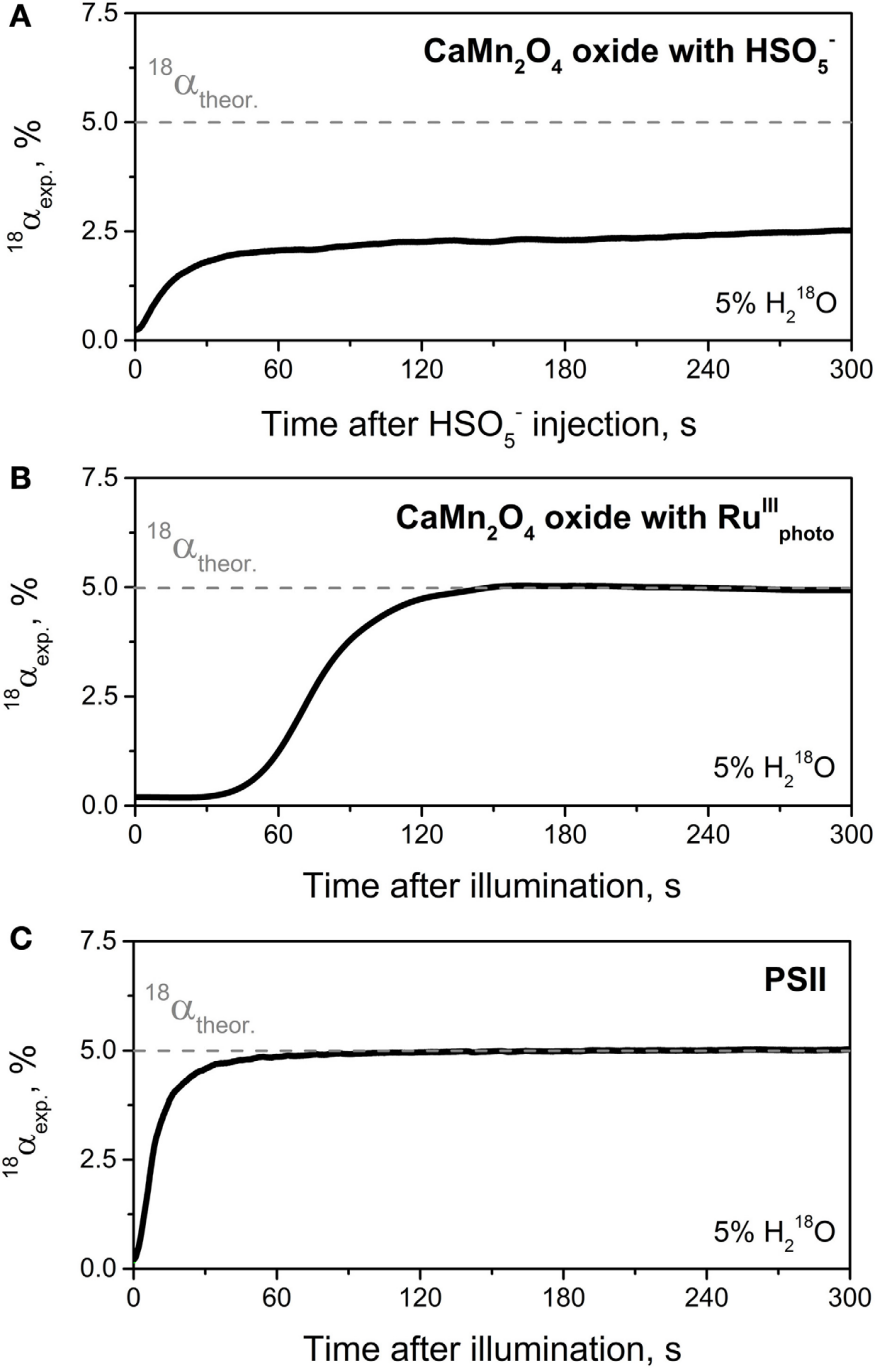
**Development of the ^18^O-isotope fraction (^18^α) over time for the course of the catalytic O_2_-formation in reactions catalyzed by synthesized CaMn_2_O_4_ · H_2_O oxide (A,B) and by the WOC of PSII (C). (A)** Change in ^18^α-value for the reaction of CaMn_2_O_4_ · H_2_O with HSO^−^_5_ (oxone) indicating that only one of the two oxygen atoms of O_2_ evolved originates from the bulk water. A solution of HSO^−^_5_ in H^18^_2_O-enriched water was injected at *t* = 0 into the MIMS cell filled in with a non-enriched oxide suspension (1 mg ml^−1^; pH ~4.5) to give a final HSO^−^_5_ concentration of 3.7 mM and an H^18^_2_O enrichment of 5%. Note that the rise of ^18^α to the value of 2.5% corresponds to half the percentage of the ^18^O-labeled water. **(B)** Change in ^18^α-value for the reaction of CaMn_2_O_4_ · H_2_O with photogenerated [Ru^III^(bipy)_3_]^3+^. Shortly before illumination (started at *t* = 0) the reaction mixture (H^18^_2_O (5%), CaMn_2_O_4_ · H_2_O (1 mg ml^−1^), [Ru(bipy)_3_]^2+^ (1.5 mM), and [Co(NH_3_)_5_Cl]^2+^ (12.5 mM); pH ~4) inside the MIMS cell was purged with N_2_ until “zero” O_2_ level was reached. **(C)** Change in ^18^α-value for O_2_ production by PSII membrane fragments isolated from spinach. O_2_ evolution was induced by actinic continuous light at *t* = 0. Other conditions: 5% H^18^_2_O, [Chl] = 0.03 mg ml^−1^, 0.6 mM PPBQ, 2 mM K_3_[Fe(CN)_6_], pH 6.0, and 20°C. Gray dashed lines in all panels indicates the theoretical ^18^α value expected for reaction of the “true” water-splitting, i.e., when both oxygen atoms of formed O_2_ originate from water. In all cases O_2_ production was detected by TR-MIMS as ^16^O_2_ (at *m/z* 32), ^16^O^18^O (*m/z* 34), and ^18^O_2_ (*m/z* 36), and the ^18^α was calculated according to the following equation: ^18^α = ([^18^O_2_] + 1/2[^16^O^18^O])/[O_2_]_total_. Adapted from Shevela et al. (2011b).

One of the central goals of artificial photosynthesis is the development of bio-inspired, efficient and robust catalysts that are able to split water employing the energy of sunlight in a fashion similar to the water-oxidizing Mn_4_CaO_5_ cluster in PSII (Concepcion et al., 2012; Nocera, 2012; Wiechen et al., 2012). Therefore, data concerning catalytic rates and turnover numbers (stability) of newly synthetized O_2_-evolving catalysts are highly important for their further development. In this regard, in addition to traditionally used amperometric methods for O_2_ detection (Renger and Hanssum, 2009), TR-MIMS can be applied as a highly sensitive method for studying the O_2_-evolving capability of these complexes. However, a major advantage and uniqueness of the TR-MIMS technique in this field is that, in combination with ^18^O-labeling experiments, it can be employed for studying the pathways of O_2_ formation in reactions catalyzed by the ‘potential’ solar water-oxidation catalysts (Poulsen et al., 2005; Beckmann et al., 2008; Sala et al., 2010; Shevela et al., 2011b; Najafpour et al., 2012; Vigara et al., 2012). Thus, TR-MIMS detection of the isotopologues of O_2_ (^16^O_2_, ^16^O^18^O, ^18^O_2_) during catalytic O_2_-formation in the ^18^O-enriched aqueous solutions allows to analyze the ^18^O-fraction (^18^α) of the evolved O_2_ with good time resolution and very high accuracy. A correlation of the ^18^O-fraction in the substrate water (^18^α_theor_; reflects the H^18^_2_O-enrichment of the solvent water) and in the product O_2_ (^18^α_exp_) gives important information about the origin of the O atoms in the produced molecular oxygen. For instance, the incorporation of exactly half of the possible ^18^O-fraction into the evolved O_2_ may indicate that only one of the two O atoms of the O_2_ product originates from the bulk water as it has been monitored by TR-MIMS in the reactions of O_2_-evolving catalysts with oxygen-transferring oxidizing agent, oxone (HSO^−^_5_) (Poulsen et al., 2005; Beckmann et al., 2008; Shevela et al., 2011b) (see Figure 6A). In the case of “true” water-splitting, ^18^O-fractions in bulk water and in evolved O_2_ are expected to be same (i.e., ^18^α_theor_ = ^18^α_exp_) as depicted in Figure 6B for the reaction of a synthetic catalyst (CaMn_2_O_4_ · H_2_O oxide) with photogenerated oxidizing agent [Ru^III^(bipy)_3_]^3+^ (Ru^III^_photo_), and in Figure 6C for natural light-induced water-splitting reaction performed by PSII. It's worth mentioning here, that the initial phase of the presented traces until stable ^18^α values (Figure 6) is a technical artefact, merely caused by the response time of the membrane-inlet system of the mass spectrometer which seems to be related to the overall O_2_ concentration. However, the difference in time needed to reach final ^18^α value in two water-splitting reactions shown in Figure 6 also reflects a much slower reaction rate for the reaction of the oxide with Ru^III^_photo_. Thus, O_2_ evolution for this reaction was detected only after 1 min of illumination since this time is required to build up a sufficient concentration of photosensitizer Ru^III^_photo_ (data not shown here; for details, see Shevela et al., 2011b). We note that one of the attractive extensions to the described TR-MIMS approach for the characterization of water-splitting catalysts is the coupling of the TR-MIMS instrument to an electrochemical cell (for further details, see Konermann et al., 2008 and refs therein).

## Conclusions and future perspectives

Application of the TR-MIMS technique and isotope labeling for studies of various biophysical aspects of photosynthetic water-splitting and O_2_ production is continuously growing. It provides not only insightful and unique information (which is sometimes not accessible by other methods) about this fundamental biological process, but also becomes an essential and highly precise tool for testing artificial water-oxidizing catalysts. Future applications of TR-MIMS for studies of water-splitting chemistry and O_2_ production could follow from advances in membrane materials, different designs of the membrane-inlet systems, coupling with electrochemistry and spectroscopy, and technological developments of the mass spectrometers.

### Conflict of interest statement

The authors declare that the research was conducted in the absence of any commercial or financial relationships that could be construed as a potential conflict of interest.

## Abbreviations

C_i_: inorganic carbon (CO_2_, H_2_CO_3_, HCO^−^_3_, CO_3_^2−^)
Chl: chlorophyll
PSII: photosystem II
RC: reaction center
WOC: water-oxidizing complex.
